# Draft Genome Sequence of Rifamycin Derivatives Producing *Amycolatopsis mediterranei* Strain DSM 46096/S955

**DOI:** 10.1128/genomeA.00837-14

**Published:** 2014-08-14

**Authors:** Priya Singh, Rashmi Kumari, Udita Mukherjee, Anjali Saxena, Utkarsh Sood, Rup Lal

**Affiliations:** Department of Zoology, University of Delhi, Delhi, India

## Abstract

*Amycolatopsis mediterranei* DSM 46096 produces antibiotics of the rifamycin family, 27-demethoxy-27-hydroxyrifamycin B, 25-desacetyl-27-demethoxy-27-hydroxyrifamycin, and 27-demethoxy-27-hydroxyrifamycin SV, which are effective against Gram-negative bacteria. Here, we present the draft genome of *A. mediterranei* 46096 (approx. 10.2 Mbp) having 104 contigs with a GC content of 71.3% and 9,382 coding sequences.

## GENOME ANNOUNCEMENT

Rifamycins are produced commercially by several strains of *Amycolatopsis mediterranei*. Semisynthetic derivatives of rifamycin are used for curing tuberculosis and leprosy. There is ambiguity about the origin of these strains, which moved from one industry to another during the past 50 years. In an attempt to further analyze these strains we initially did the taxonomical characterization (1) and then sequenced the genomes of *A. mediterranei* S699 (2), 46095 (3), and 40773 (4). One strain of *A. mediterranei* that originated during mutagenesis of wild-type *A. mediterranei* 43304 (ME/83) (5) with *N*-methyl-*N*-nitro-*N*-nitrosoguanidine at Lepetit laboratory, is *A. mediterranei* 46096. Its significance lies in its ability to produce antibiotics of the rifamycin family, 27-demethoxy-27-hydroxyrifamycin SV, 27-demethoxy-27-hydroxyrifamycin B, and 25-desacetyl-27-demethoxy-27-hydroxyrifamycin, which are effective against Gram-negative bacteria (6).

Due to the severe problem of tuberculosis and the need for the production of analogues of rifamycin, there is a renewed interest in research on *A. mediterranei*. While during the past 30 years emphasis has been on the development of cloning vectors and a transformation system for this group of organisms (7–11) and on the characterization of the *rif*PKS gene cluster (12–14), the genomes of some of strains, including S699 (2), 46095 (3), 40773 (4), U32 (15), and RB (CP003777), have recently been sequenced. We report here the draft genome sequence of *A. mediterranei* 46096.

*A. mediterranei* 46096 genomic DNA was sequenced by Illumina Genome Analyzer IIx, which generated approximately 2.3 Gb of data (500 bp and 2 kb paired-end libraries) using a PCR-free approach. Sequence coverage of more than 100× was obtained, corresponding to 25,763,590 pair-end reads. Raw reads were assembled into contigs (*n* = 104, >500 bp) using ABySS software version 1.3.5 (16), set at a k-mer length of 63. The assembled genome had *N*_50_ value of 462 kb and an average GC content of 71.3%. The draft genome was annotated using RAST version 4.0 (17) and NCBI Prokaryotic Genome Annotation Pipeline (PGAP) version 2.1 (http://www.ncbi.nlm.nih.gov/genomes/static/Pipeline.html), which identified 9,382 protein-coding genes and 24 pseudogenes. Using PGAP annotations, 15 rRNA and 52 tRNA genes were also predicted. Using antiSMASH (Antibiotic and Secondary Metabolites Analysis Shell) (18), 37 secondary metabolite gene clusters were found that encode 7 type I and II polyketide synthases (PKS), 12 nonribosomal peptide synthetases (NRPSs), 2 NRPS/PKS, lantipeptides, bacteriocin, etc. The rifPKS gene cluster was identical to the earlier reported rifPKS (12), except for the *rif*B gene, which was 99% similar. Additionally, one CRISPR element and 1,212 tandem repeats were also identified in the draft genome. Average nucleotide identity (ANI) (19) revealed that *A. mediterranei* 46096 is highly similar to *A. mediterranei* U32 (99.9%) (15), S699 (99.9%) (2), RB (CP003777), and 40773 (approx. 99.9%) (4) but substantially differs from 46095 (approx. 92.5%) (3).

With the availability of the genome sequences of the above-mentioned strains of *A. mediterranei*, including DSM 46096, comparative genomics studies are now under way to better understand the variations in polyketide synthase gene clusters and related genome characteristics. The analysis will further resolve the issue of origin of these strains hitherto unknown and to perform combinatorial biosynthesis for the production of rifamycin analogues (20).

### Nucleotide sequence accession numbers.

The draft genome sequence of *A. mediterranei* DSM 46096 is available in GenBank database under accession number JMQG00000000. The version described in this paper is JMQG01000000.
